# Microneedle mediated transdermal delivery of β-sitosterol loaded nanostructured lipid nanoparticles for androgenic alopecia

**DOI:** 10.1080/10717544.2022.2120927

**Published:** 2022-09-15

**Authors:** Kousalya Prabahar, Ubaidulla Udhumansha, Nehal Elsherbiny, Mona Qushawy

**Affiliations:** a Department of Pharmacy Practice, Faculty of Pharmacy, University of Tabuk, Tabuk, Saudi Arabia; b Department of Pharmacy Practice, Faculty of Pharmacy, Dr. M.G.R. Educational and Research Institute, Velappanchavadi, Chennai, Tamil Nadu, India; c C.L. Baid Metha College of Pharmacy, Chennai, India; d Department of Pharmaceutical Chemistry, Faculty of Pharmacy, University of Tabuk, Tabuk, Saudi Arabia; e Department of Biochemistry, Faculty of Pharmacy, Mansoura University, Mansoura, Egypt; f Department of Pharmaceutics, Faculty of Pharmacy, University of Tabuk, Tabuk, Saudi Arabia; g Department of Pharmaceutics, Faculty of Pharmacy, Sinai University, Alarish, North Sinai, Egypt

**Keywords:** Androgenic alopecia, β-sitosterol, nanostructured lipid carrier, polymeric microneedles, chitosan, testosterone induced alopecia

## Abstract

Plant-derived 5 α-reductase inhibitors, such as β-sitosterol and phytosterol glycosides, have been used to treat androgenic alopecia, but their oral absolute bioavailability is poor. This study aimed to develop a transdermal drug delivery system of β-sitosterol (BS) using a nanostructured lipid carrier (NLC) incorporated into polymeric microneedles (MN). Using a high-speed homogenization method, NLC was formulated variables were optimized by Box-Behnken statistical design. The optimized formulation of BS-loaded NLCs was incorporated into the chitosan-based MNs to prepare NLC-loaded polymeric MNs (NLC-MNs) and evaluated using testosterone induced alopecia rats. The cumulative amount of β-sitosterol associated with NLC- MN which penetrated the rat skin in-vitro was 3612.27 ± 120.81 μg/cm^2^, while from the NLC preparation was 2402.35 ± 162.5 μg/cm^2^. The steady state flux (J_ss_) of NLC-MN was significantly higher than that of the optimized NLC formulation (*P* < 0.05). Anagen/telogen ratio was significantly affected by NLC and NLC-MN, which was 2.22 ± 0.34, 1.24 ± 0.18 respectively compared to 0.26 ± 0.08 for animal group treated with testosterone. The reversal of androgen-induced hair loss in animals treated with β-sitosterol was a sign of hair follicle dominance in the anagenic growth phase. However, NLC–MN delivery system has shown significant enhancement of hair growth in rats. From these experimental data, it can be concluded that NLC incorporated MN transdermal system have potential in effective treatment of androgenic alopecia.

## Graphical Abstract

**Figure:**
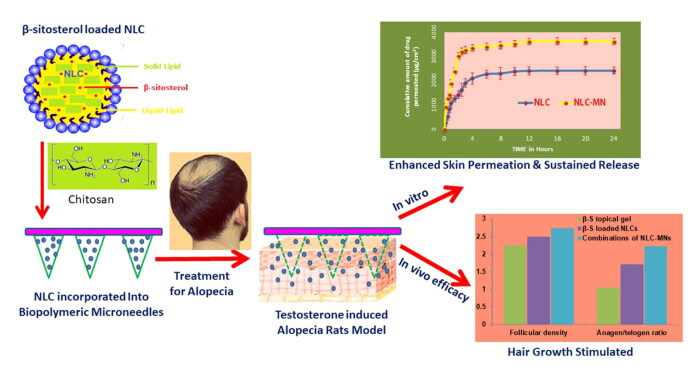

## Introduction

Alopecia is the partial or total reduction of hair in a specific area of the skin that affects millions of men and women worldwide (Pereira et al., 2018). Alopecia affects 50% and 40% of adult male and female respectively, and the percentage is increasing by nearly 5% every year (Ashique et al., 2020). Androgenetic alopecia has been linked to higher levels of dihydrotestosterone (DHT) in balding scalp follicles than in non-balding ones. Hair loss is caused by an increase in the concentrations of 5α-reductase and androgen receptors. Androgenetic alopecia was first treated with finasteride, a 5-hydroxysteroid reductase inhibitor, which was approved by the FDA to be used in the handling of alopecia. As a result, it can cause impotence and other sexual dysfunctions as well as testicular pain and myalgia (Jain et al., 2015). Therefore, development of alternative safe and effective therapeutic strategies is of great demand. Natural products have been widely used to treat androgenic alopecia (AGA) due to their safety with less or no toxic effects.

β-Sitosterol is a natural phytosterol that presents in various parts of plants like leaves, fruits, and rhizomes. Studies have reported that β-Sitosterol has many pharmacological activities like angiogenic, antioxidant, immunomodulatory, antimicrobial, antidiabetic, anti-inflammatory, and anticancer (Salen et al., 1970; Prager et al., 2002). Interestingly, β-sitosterol has been used for the treatment of AGA due to 5α-reductase inhibiting activity (Cabeza et al., 2003). The main challenges associated with the oral administration of β-sitosterol is poor water and oil solubility, crystalline nature at ambient and body temperatures, and high daily doses (i.e. up to 3 g/day). Complexation with cyclodextrin, liposomal, electrospun nanofibers, solid lipid nanoparticles, self-emulsion drug delivery system (SMDDS), and nanostructured lipid carriers (NLCs) have all been studied to improve therapeutic efficacy of by improving β-sitosterol oral absorption (Zhang et al., 2020). A really challenging fact is to successfully incorporate β-sitosterol in the drug preparations.

The topical application of β-sitosterol has received much attention for the treatment of AGA. Upadhyay et al. (2012) developed Phyto-vesicles loaded β-sitosterol by the process involving its complexation with phosphatidylcholine and found that β-sitosterol has increased absorption and improved activity in alopecia treatment (Upadhyay et al., 2012). Limitation of phospholipid vesicles is leakage and fusion of encapsulated drug, oxidation of phospholipid, high cost, low solubility, and poor stability. Due to the lack of an aqueous core, NLCs have a higher entrapment effectiveness for hydrophobic pharmaceuticals than lipid bilayers like liposomes. It contains a stiff core lipid matrix that improves drug stability, repeatability, and large-scale synthesis while avoiding the use of organic solvents. Virgin coconut oil (VCO), which has a lot of potential for topical applications is employed as a liquid lipid in the synthesis of NLCs in this study to improve the stability and skin penetration ability. However, because of the stratum corneum (SC), NLC transfer across the skin remains a significant challenge.

To overcome this limitation, the present study aimed at development of polymeric microneedles (MN) for sustained release of β-sitosterol, thereby achieving prolonged plasma drug concentrations for efficient treatment of AGA. In recent years, advancement of microneedle technology in biomedical applications has made significant progress in various fields like vaccine delivery and cosmeceuticals, diagnosis and treatment of the diseases (Prausnitz, 2004). USFDA has recently issued guidance on ‘Regulatory Considerations for Microneedling Products’, creating a lot of scope for research in biomedical applications of microneedles (Gorantla et al., 2021). MNs have several advantages, including the ability to pierce the stratum corneum barrier, making them a self-administerable, painless alternative to hypodermic needles for bolus medication delivery. They provide a long-term drug delivery system for treating disorders like AGA. Despite the fact that metal microneedles have a better insertion ability and increased drug administration, they must be thrown as biohazardous sharp waste after usage (Chu & Prausnitz, 2011). Dissolving microneedles which made of water-soluble polymers such as polyvinylpyrrolidone (PVP), hyaluronic acid (HA), and carboxymethyl cellulose (CMC) are difficult to fully implant into the skin due to their wide needle shape and low mechanical strength (Bariya et al., 2012).. If the microneedles are inserted incompletely, it will result in inefficient drug delivery and wastage of medication.

Because of their high biocompatibility, biodegradability, and nontoxic qualities polymeric MNs have gotten a lot of attention. Polymeric MNs do not leave any sharp biohazard medical waste after use (Lee et al., 2013). Polymers can be fabricated with varying swelling properties and degradation profiles to varying mechanical functions and properties. PLGA, Chitosan, and its derivatives have been used for the preparation of MNs which can enhance the therapeutic potential of encapsulated drugs (Al-Japairai et al., 2020).

However, incorporating many lipophilic drugs into these hydrophilic polymeric MNs is very difficult. A particle-based MN system with antigen trapping in a polymeric nanoparticle was designed to release the antigen into the skin in a prolonged manner. The nanoparticles allow the release of entrapped drug in a controlled manner after releasing from the MN to the dermal layer, resulting in a long-acting effect at the site of action. Lipid-based particulate carriers, such as solid lipid nanoparticles (SLNs) and natural lipid nanoparticles (NLCs) have been introduced for the formulation of lipophilic drugs into water-soluble polymeric matrices because these drug-loaded nanoparticles can be dispersed homogeneously in an aqueous phase (Lee et al., 2014; Guo et al., 2018). A lot of studies were done to find effective solution for alopecia. Ushirobira et al. (2020) developed poly-(ɛ-caprolactone)-lipid-core nanocapsules (NC) containing duasteride for targeting the hair follicles and the results showed increase in the drug accumulation in hair follicle by 5 fold compared to the drug solution (Ushirobira et al., 2020). Oliveira et al. (2020) prepared a Poly-ε-caprolactone nanocapsules loading latanoprost for topical treatment of alopecia by nanoprecipitation of the polymer on the surface of drug oily nanodroplets and they found that Poly-ε-caprolactone nanocapsules delivered 30% more drug to these skin structures relative to the control solution of the drug (Oliveira et al., 2020).

The goal of the study was to create biodegradable chitosan-based polymeric MNs for long-acting administration of encapsulated β-sitosterol with NLCs. The chitosan MNs could act as a reservoir, which control the release of entrapped β-sitosterol. The swelling and diffusion mechanisms, as well as the progressive disintegration of the chitosan MNs, could be used to produce this protracted release (Gorantla et al., 2021). Sustained transdermal delivery will minimize the dermatitis caused by long-term use of transdermal patches or adhesives. From our best knowledge, no reports are available regarding chitosan MNs loaded β-sitosterol for treating alopecia. β-sitosterol encapsulated Chitosan MNs were developed and characterized by FTIR, DSC, scanning electron microscope (SEM) and drug content were also evaluated. Skin penetration ability, in-vitro drug release, ex-vivo skin permeation study and stability were investigated. The in-vivo transdermal delivery of NLC-MN of β-sitosterol in testosterone-induced alopecia in rats was evaluated.

## Materials and methods

β-sitosterol and Chitosan were purchased from Sigma Aldrich, USA. Glyceryl mono stearate and virgin coconut oil were received as a gift from Masterowin Pharmaceuticals, India. Sodium hydrochloride, Potassium Dihydrogen Phopsphate and acetic acid were bought from Merck, India. Distilled water was obtained from inner source. The reagents and chemicals of analytical grade were used in this study.

All other chemicals and reagents used in the study were of analytical grade.

### Optimization of β-sitosterol loaded NLCs

Using design-expert software version 11.0, a Box-behnken statistical design with three factors, three levels, and seventeen runs was used for the optimization investigation. In our early investigations, the variables stirring speed, lipid concentration, and drug concentration were the most critical factors that influenced particle size, entrapment efficiency %, and the in-vitro drug release of NLCs. Based on the preliminary trial results, stirring speed (A), lipid concentration (B), and drug concentration (C) were chosen as independent variables and the values were established at high, medium, and low. Seventeen NLC formulations were produced and characterized for particle size (R1), entrapment efficiency (R2), and drug release according to the design (R3). Initially, we conducted tests with a variety of experimental ranges. Based on these findings, we changed the range values for each element in our study. Based on the formulation characteristics, this design examined the main, interaction, and quadratic effects of the independent variables.

### Preparation of β-sitosterol loaded NLCs

The β-sitosterol loaded NLC was prepared by using high speed homogenization method (Patel & Patel, 2021). The lipid phase was first created by heating Glyceryl mono stearate (solid lipid) and virgin coconut oil (liquid lipid) to 750 degrees Celsius. The lipid phase mixture was melted to create a clear and homogenous lipid mixture. β-sitosterol was added to the lipid mixture once it had completely melted. On the other hand, an aqueous phase was created by extensively mixing distilled water and Tween 80. The aqueous mixture was then heated to 500 °C before being dropped into the lipid phase and stirred continuously at 750 rpm to generate a pre-emulsion mixture. To prevent lipid crystallization, a pre-emulsion was homogenized using a Remi motor RQ 134H at 10000 rpm for 10 minutes, followed by an ultrasonication treatment for 6 minutes. The obtained O/W emulsion was congealed to room temperature with continuous stirring where the lipid phase was then recrystallized into NLCs (Qushawy, 2021). The NLC dispersions were stored in well closed container and used for further studies.

### Characterization of NLCs

#### Determination of the particle size (Y1)

The mean particle size of NLC formulations was estimated using Malvern® Zetasizer Nano ZS90 (Malvern^®^ Instruments Limited, Worcestershire, UK). After diluting with distilled water (1:200), triplicate measurements were made using a 90° scattering angle **(**Elmowafy et al., 2017 **)**.

### Determination of the entrapment efficiency % (Y2)

The drug encapsulation efficiency (EE) was measured using an indirect approach in which an aliquot (2 ml) of NLCs was centrifuged for 45 minutes at 7000 rpm (Elmowafy et al., 2018). A reverse phase high performance liquid chromatography (RP HPLC) equipment was used to determine the amount of un-encapsulated medication in the clear supernatant fluid (Singh et al., 2019). HPLC was performed on an RP18 column with a 20 µl injection volume and a mobile phase of methanol and acetonitrile (30:70 v/v). The samples were eluted at a flow rate of 1 mL/min while the eluent was measured at a wavelength of 210 nm. The following equation was used to compute the encapsulation efficiency of β-sitosterol.

EE% = (Total drug−Un−entrapped drug/Total drug) × 100

### In-vitro drug release study (Y3)

An in-vitro drug release study of the β-sitosterol from the prepared NLC formulations was performed using the dialysis bag method. Dialysis membranes (cut-off 14,000 Da) were cleaned and soaked in the dissolution medium overnight. The bags were filled with a β-sitosterol-laden NLC suspension, which was then placed into dialysis bags and submerged in a 50 ml phosphate buffer (pH 7.4) containing 2.5% Tween 80 at 37 ± 1 °C stirred at 100 rpm (Soleimanian et al., 2020). The samples were taken at predetermined time intervals and the amount of β-sitosterol released was measured using the HPLC technique.

To examine the release kinetic pattern, the in-vitro drug release data was fitted with various mathematical models, including zero, first, Higuchi, and Korsmeyer – Peppas. The Korsmeyer–Peppas model provided the greatest match for NLC formulation curves based on the coefficient of determination (R2):

$$Q\;=\;k\cdot t^{n}$$

where Q = the proportion of β-sitosterol released at time (t), k = the release rate constant, and n = the release exponent that typifies the drug release mechanism: Fickian diffusion is indicated by *n* = 0.43, zero-order release is indicated by *n* = 1, while 0.43 < *n* < 1 values are related to anomalous transport.

### Optimization of formulation factors

The optimization process was done by Box-Behnken design, using Design-Expert 11 software, to determine the optimum level of the formulation factors which obtain NLC formulation with the desired responses. The goal of the optimization process was to develop an optimized formulation with minimum particle size, maximum EE (%), and prolonged drug release Q6h (%).

### Drug loading capacity of the optimized formulation

To determine β-sitosterol loading in the prepared optimized formulation of NLC, 10 µL of the optimized NLC formulation was mixed with 1 mL methanol followed by sonication using water bath sonicator for 10 minutes at 40 °C to dissolve the β-sitosterol from the lipid carriers (Lee et al., 2014). The following equation was used to calculate the drug loading capacity (mg βS/g lipid):

DL = (R – C)/L

where R is the total amount of drug remaining in the NLC dispersion, C is the amount of drug detected in the methanol phase, and L is the total amount of lipid added.

### Determination of the surface morphology of the optimized formulation by scanning electron microscopy (SEM)

The shape and surface morphology of the optimized NLC was determined by SEM. The optimized formulation of β-sitosterol NLC was dusted on a double-sided tape of aluminum stub. The sample was coated with gold sputter coater to a thickness of 400 A^0^. The gold coated sample was scanned at 0.6 mmHg chamber pressure and 20 kV accelerated voltage (Gaba et al., 2015).

### Fabrication of polymeric microneedles

To produce 2% (w/v) polymeric solution, chitosan polymer was dispersed to be dissolved in (1% v/v) an acetic acid aqueous solution (Castilla-Casadiego et al., 2021). The polymeric solution was dialyzed against deionized water at a temperature of 37 ± 0.5 °C using a dialyzing tube (cut-off size 14 k Dalton). The resultant polymeric solution was dried in a water bath (37 °C) till the chitosan solution becomes 10% w/v (Arshad et al., 2019). Chitosan 10% w/v solution was casted onto the polydimethylsiloxane pre-fabricated microneedle molds (10 × 10 cavities) with a depth of 300 µm. The molds were subjected to centrifugation at 1000 rpm for 5 min to fix the solution of chitosan into the mold cavities. After that, mold was transferred into the positive pressure chamber and then the pressure was released gradually. Then the silicone molds were dried at 37 °C for 24 h. MNs were removed from the molds and evaluated for successful needle formation by visual inspection. To fabricate NLC-MNs, 10 mL of the optimized formulation was mixed with chitosan solution, and a similar method was adopted to prepare microneedles.

## Evaluation of NLC-MNs

### Morphological studies

By observing through a microscope, tip diameter, base diameter, and the length were determined for seven arrays of NLC-MNs. The length to base diameter ratio gives the aspect ratio (Lee et al., 2014). All determinations were done in triplicates and Mean ± SD were calculated.

### Drug content

An array of NLC-MNs was allowed to be dissolved in distilled water containing 2.5% Tween 80 by stirring at 300 rpm using magnetic stirrer for 1 hr. the resultant solution was mixed with methanol for dilution and subjected to sonication for 5 min to allow complete dissolution of β-sitosterol from fabricated NLC-MNs (Rojekar et al., 2021). The drug content in the prepared MNs was determined by analysis using HPLC.

### Scanning electron microscopy of NLCs-MN

The microneedle patches morphology was examined for needle shape, size, and other physical characteristics using SEM. The samples were coated with gold solution operating at 10 kV for 15 s under low vacuum to obtain a better contrast (Arshad et al., 2021).

### In-vitro permeation studies of NLCs-MN

In-vitro permeation experiments were conducted for NLC-MNs formulations using Franz diffusion cell (Serpe et al., 2019). Male Wistar rats weighing 200 to 250 g with full-thickness abdominal skin were used. The hair of the abdominal area was shaved carefully using an electric clipper. The skin was thoroughly cleaned from any adhering tissues or blood vessels with distilled water. Then, the skin was soaked in phosphate buffer pH 7.4, for 1 hr, before beginning the experiment. The rat skin piece was isolated, and placed between the donor and receptor compartment of Franz diffusion cells where the epidermis faced upward while the dermis layer was downward (Pamornpathomkul et al., 2017). Transdermal microneedle patch formulations (NLC-MN) were applied to rat skin. To ensure a prompt mass transfer, the recipient compartment solution was stirred at 600 rpm following permeation (Courtenay et al., 2018). The diffusion medium was kept at 37 ± 0.5 °C, stirred at 100 rpm, and provided with Tween 80 (2.5%) to ensure the sink condition. At predetermined time intervals, 1 ml sample was withdrawn from the receptor compartment and replaced with an equal volume of fresh medium. The β-sitosterol concentration present in the sample was analyzed by HPLC and the cumulative amount of permeated drug was plotted against times. The steady state flux (Jss) of the NLC and NLC-MN was determined from the slope of the linear portion of the cumulative drug release plots (Serpe et al., 2019).

### Animals

Male wistar rats weighing 230–250 g were obtained from the C.L. Baid Metha College of Pharmacy’s Central Animal House in Chennai, India. The animals were housed in a typical laboratory setting with a temperature of 25 °C and a relative humidity adjusted at 55%. The animals were housed in polypropylene cages that were separated into four groups of four animals each. They receive unlimited water and eat a standard laboratory diet (Lipton feed, Mumbai, India). The Institutional Animal Ethical Committee authorized the experimental protocol (IAEC Approval no: 04/321/PO/Re/S/01/CPCSEA).

### Animal groups and treatments

The method reported by Upadhyay et al. was followed with minor changes (Upadhyay et al., 2012). Five groups of animals, each with six to eight animals, were prepared. Depilatory cream was used to remove the dorsal hair of mice (2 by 2.5 cm). The following animal groups were given different treatments: (A) Intact control (did not receive testosterone) (B) Testosterone solution only; (C) Testosterone with β-sitosterol gel; (D) Testosterone + NLCs and (E) Testosterone + NLC-MNs, the patch was firmly pressed for 1st 30 seconds to penetrate through the epidermis and the pressed softly for extra 2 min. The patch base was peeled at 10 min post insertion into the skin, allowing the settled MNs in the skin for further sustained drug release. Except group A, all other groups were administered testosterone (0.5 mg/Kg/Daily) subcutaneously (SC) and Group C, D, and E received β-sitosterol (1 mg/Kg/Daily) topically for 21 days.

### Effect of NLC-MN on hair growth of the rats

The hair growth was observed by qualitative evaluation. After 21 days, the difference in hair growth in each group was examined visually and photographed. A quantitative analysis of hair growth was performed. After shaving the long hair, the animal skin in the dorsal area was dissected and fixed in 10% formalin (Dhanotia et al., 2011). Vertical sections of the skin were prepared after fixation and stained with hematoxylin and eosin (H&E). To evaluate hair growth, the sections were analyzed for various parameters. The follicular density (number of follicles/mm) was reported by recording the number of hair follicles in 2 mm area. Anagen/telogen ratio was determined by calculating the number of follicles in anagen phase (active growth phase) and telogen phase (resting phase) (Noubarani et al., 2014).

### Skin irritation test

One day before this stage of experiment, the hair on the dorsal area was removed (Dudhipala et al., 2020). The rats were divided into 4 groups with 6 in each group. Group I acted as control, group II received NLC-MN without drug, group III received NLC-MN with β-sitosterol, and group IV received a standard irritant with 0.8% v/v formalin aqueous solution (Ahmad et al., 2018). NLC-MN, or new formalin solution, was applied daily for 7 days. Finally, the same investigator grades the application sites according to a visual scoring scale (Al-Kasasbeh et al., 2020).

### Statistical analysis

Data was reported as mean ± SD. Data was analyzed by one-way analysis of variance (ANOVA) followed by Tukey’s post hoc test. *P* < 0.05 was considered as significant.

## Results and discussion

β -sitosterol-loaded NLC is developed particularly for topical use since the nanosize ensures intimate contact with the skin and controls the drug release. NLC was mainly used as a carrier for lipophilic molecules due to its high affinity to the lipid phase resulting in a high loading capacity.

In this study, Box-behnken statistical design was employed for the optimization study using design-expert software version 11 to study the effect of the formulation factors in the responses. Three independent variables were employed, stirring speed (A), lipid concentration (B), and drug concentration (C). Each independent variable was set at high, medium, and low levels on the basis of the results of initial trials, Table 1.

**Table 1.** Independent variables and their constraints of β-sitosterol-loaded NLCs.

Variables (Independent variables)	Constraints		
	−1	0	+1
A- Stirring speed (rpm)	5000	7500	10000
B- Lipid concentration (% )	70	80	90
C- Drug concentration (%)	2.5	5	7.5
Response (dependent variables)	Goal		
R1-Particle size (nm)	Minimize		
R2-Entrapment efficiency (%)	Maximize		
R3-Drug release (%)	Minimize		

Seventeen NLC formulations were produced using the Box-behnken statistical design, with the composition shown in Table 2. Particle size (R1), entrapment efficiency (R2), and drug release (R3) were all measured in the developed NLC formulations.

**Table 2.** The designed β-sitosterol-loaded NLCs according to Box-behnken experimental design and their calculated responses.

Run	Factor 1	Factor 2	Factor 3	Response 1	Response 2	Response 3
	A:Stirring Speed (rpm)	B:Lipid (%)	C:Drug (%)	Particle Size (nm)	Entrapment Efficiency (%)	Drug Release (Q_6h_) (%)
NLC1	10000	80	2.5	150 ± 1.25	81.40 ± 1.28	53.12 ± 0.95
NLC 2	7500	80	5	210 ± 2.36	48.50 ± 2.54	50.39 ± 0.46
NLC 3	7500	80	5	230 ± 1.25	49.51 ± 0.94	50.47 ± 0.27
NLC 4	5000	80	2.5	300 ± 4.65	76.44 ± 2.75	32.25 ± 1.05
NLC 5	7500	90	7.5	195 ± 1.90	69.90 ± 2.69	41.62 ± 0.52
NLC 6	7500	80	5	200 ± 0.93	42.66 ± 0.84	39.74 ± 0.86
NLC 7	7500	90	2.5	185 ± 1.36	72.21 ± 0.74	30.24 ± 0.61
NLC 8	5000	80	7.5	260 ± 0.96	72.56 ± 1.65	29.31 ± 0.94
NLC 9	10000	80	7.5	185 ± 2.34	92.50 ± 2.54	65.20 ± 1.63
NLC 10	7500	70	7.5	198 ± 2.18	65.82 ± 1.27	50.16 ± 1.49
NLC 11	7500	80	5	174 ± 1.85	47.21 ± 0.65	48.28 ± 1.38
NLC 12	5000	90	5	310 ± 4.25	79.21 ± 1.98	18.19 ± 0.59
NLC 13	10000	90	5	185 ± 3.21	90.10 ± 2.55	43.75 ± 0.98
NLC 14	10000	70	5	167 ± 0.84	75.32 ± 1.49	61.31 ± 1.69
NLC 15	7500	80	5	167 ± 2.95	47.52 ± 1.42	56.16 ± 1.76
NLC 16	5000	70	5	290 ± 1.63	69.32 ± 2.64	25.95 ± 0.66
NLC 17	7500	70	2.5	170 ± 0.94	46.52 ± 1.29	59.62 ± 2.09

For all three dependent variables, the quadratic model with a coefficient of correlation (R2) almost equal to 1 was found to be the best-fitting model. The quadratic mathematical model for three formulation factors is provided in the below equation:

$$Y=\;\beta_{0}+\;\beta_{1}X1+\;\beta_{2}X2+\;\beta_{3}X3+\;\beta_{12}X1X2+\;\beta_{23}X2X3+\;\beta_{13}X1X3+\;\beta_{11}X1^{2}+\;\beta_{22}X2^{2}+\;\beta_{33}X3^{2}$$

where Y = the response to be evaluated, β = regression coefficient, and X_1_, X_2_ and X_3_ = factors for A, B, and C, respectively.

### Effect of the formulation factors on the responses

Seventeen formulations of β-sitosterol NLCs were prepared and evaluated for the particle size, entrapment efficiency %, and drug release. It was found that the results of these responses were affected by the formulation factors, Table 2.

### Response surface methodology

The three-dimensional surface plots show how three separate parameters interact to affect the particle size, entrapment efficiency, and drug release of NLCs. The response surface analysis plots in three-dimensional model graphs were created using the design expert program version 11.

**Figure 1.**
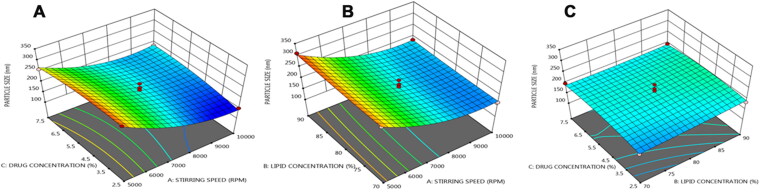
Three dimensional (3 D) response plots showing the effect of three variables on the particle size of NLCs.

Figure 1A–C demonstrate that the particle size value reduced as the stirring speed increased. This may be due to the high emulsification between the oil and aqueous phase and split the coarse emulsion into small particles in to emulsify the oil and aqueous phase and split the coarse emulsion into minute particles in presence of high stirring speed. When the stirring speed was increased, the model showed a linear increase in particle size. It was discovered that as the lipid content was increased, the size of the NLCs grew. This effect is owing to the fact that when the solid content of NLCs increases, so does the dispersion viscosity, resulting in increased surface tension and hence larger particle size (Shamma & Aburahma, 2014). This effect was further aided by increasing the surfactant concentration in the formulation, which is essential to stabilize the increased percentage of solid lipid. This is owing to the accumulation of surplus surfactant molecules on the surface of NLCs, which is mostly due to hydrophobic contact, in which nonpolar groups such as the surfactant’s alkyl chains and solid lipid molecules interact with one another (Yang et al., 2010). The quadratic equation below can be used to calculate the effect of independent factors on particle size. In the following regression equation, positive numbers before a factor indicating that the response grows as the factor increases, and vice versa.

$$\mathrm{Particle}\;\mathrm{size}\;\left(Y_{1}\right)\;=\;+196.20-59.125*A+6.25\;*B+4.125*C-0.5*\mathrm{AB}\;+18.75*\mathrm{AC}-4.5*\mathrm{BC}+39.275*A^{2}+2.525*B^{2}-11.725*C^{2}$$

**Figure 2.**
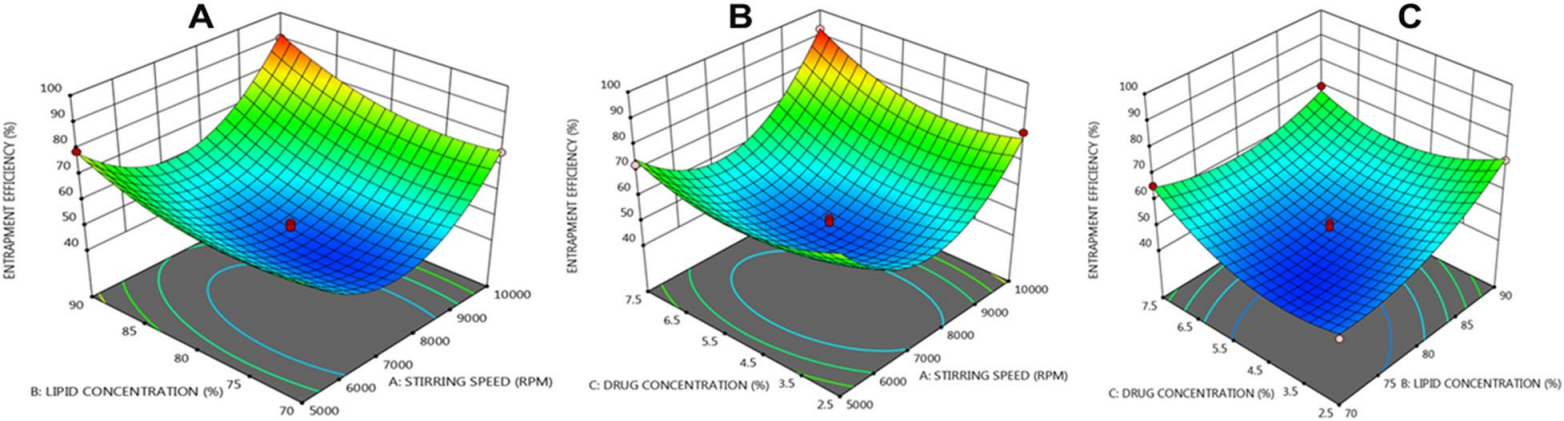
Three dimensional (3 D) response plots showing the impact of three variables on the Entrapment efficiency of NLCs.

The response surface curve of entrapment efficiency is shown in Figure 2A–C. As the lipid part of the drug was increased in proportion to the drug, more drug could be entrapped in the lipid matrix, increasing entrapment efficiency. Furthermore, increasing the volume of liquid lipids increases the solubility of medicines, resulting in higher entrapment efficiency. Maximum entrapment efficiency values were seen (red color area) as the amount of lipid concentration grew; however, lesser lipid concentration gave the lowest entrapment efficiency values of nano structured lipid. The high EE % achieved may be related to the lipophilicity of glyceryl monosterate lipids combined with crystal lattice defects that give ample space for drug β-sitosterol (Shi et al., 2013). When the lipid concentration was raised, the plotted model showed a linear increase in entrapment value. With increasing lipid concentrations, the entrapment effectiveness of nanostructured lipid carriers loaded with β-sitosterol improves. It could be related to β-sitosterol’s strong lipophilicity, which results in significant EE in lipid-based nanoparticles. Long-chain fatty acids can connect to glycerides, allowing lipophilic medicines to lodge easier, according to Dudhipala and Veerabrahma (2016). The results showed that incorporating liquid oil into lipid matrix penetrates the crystalline matrix, allowing the drug to lodge in NLC, resulting in a high EE (Woo et al., 2014; Tatke et al., 2018). High drug encapsulation efficiency in lipid nanoparticles allows for maximum drug penetration into the skin.

The quadratic equation below can be used to calculate the effect of independent variables on entrapment efficiency. In the following regression equation, positive numbers before a factor indicate that the response grows as the factor increases, and vice versa.

$$\mathrm{Entrapment}\;\mathrm{efficiency}\;\left(Y2\right)\;=+47.08+5.22375*A+6.7787*B+3.0525*C+1.22*\mathrm{AB}+3.475*\mathrm{AC}-5.35*\mathrm{BC}+\;24.286*A^{2}+7.121*B^{2}+9.3582.89*C^{2}$$

**Figure 3.**
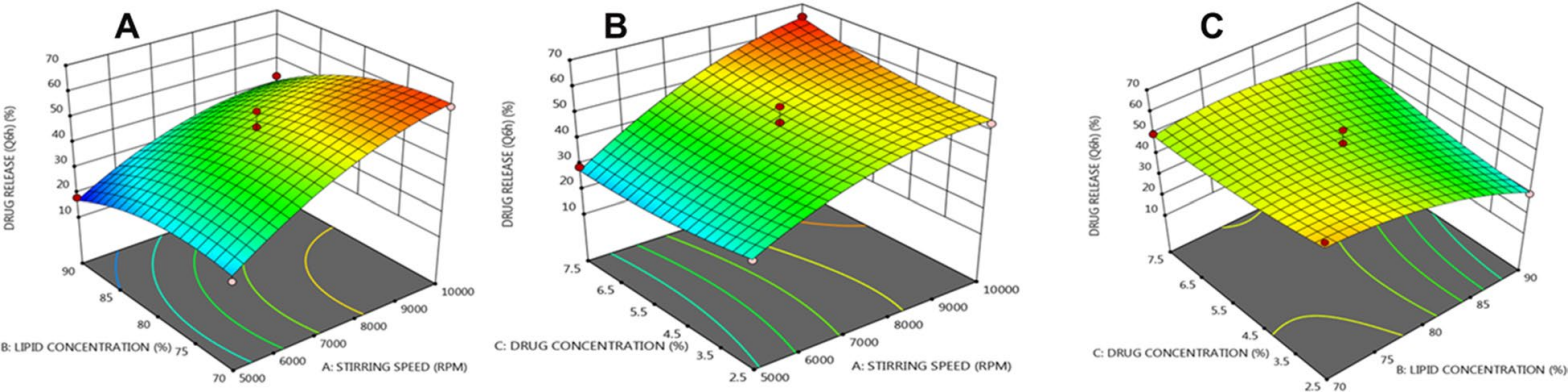
Three dimensional (3 D) response plots showing the impact of three variables on the β-sitosterol release from NLCs.

The response surface curve of drug release is shown in Figure 3A–C; the value increased as the amount of drug concentration increased, while the lowest drug release values of nanostructured lipid were observed at lower drug concentration and high lipid concentration. We discovered initial burst medication release followed by regulated release in the current investigation. The similar result was previously reported when NLC was prepared with a mixture of glyceryl monostearate and coconut oil (Azmi et al., 2020). It was found that the response increases with increasing the formulation factors and vice versa. This was indicated by the positive values before a factor in the following regression equation.

$$\mathrm{Drug}\;\mathrm{release}\;\left(Y_{3}\right)\;=\;+48.6+14.75*A-7.875*B+1.375*C-2.75*\mathrm{AB}+3.75*\mathrm{AC}+5*\mathrm{BC}-6.05*A^{2}-5.8*B^{2}+2.2*C^{2}$$

**Figure 4.**
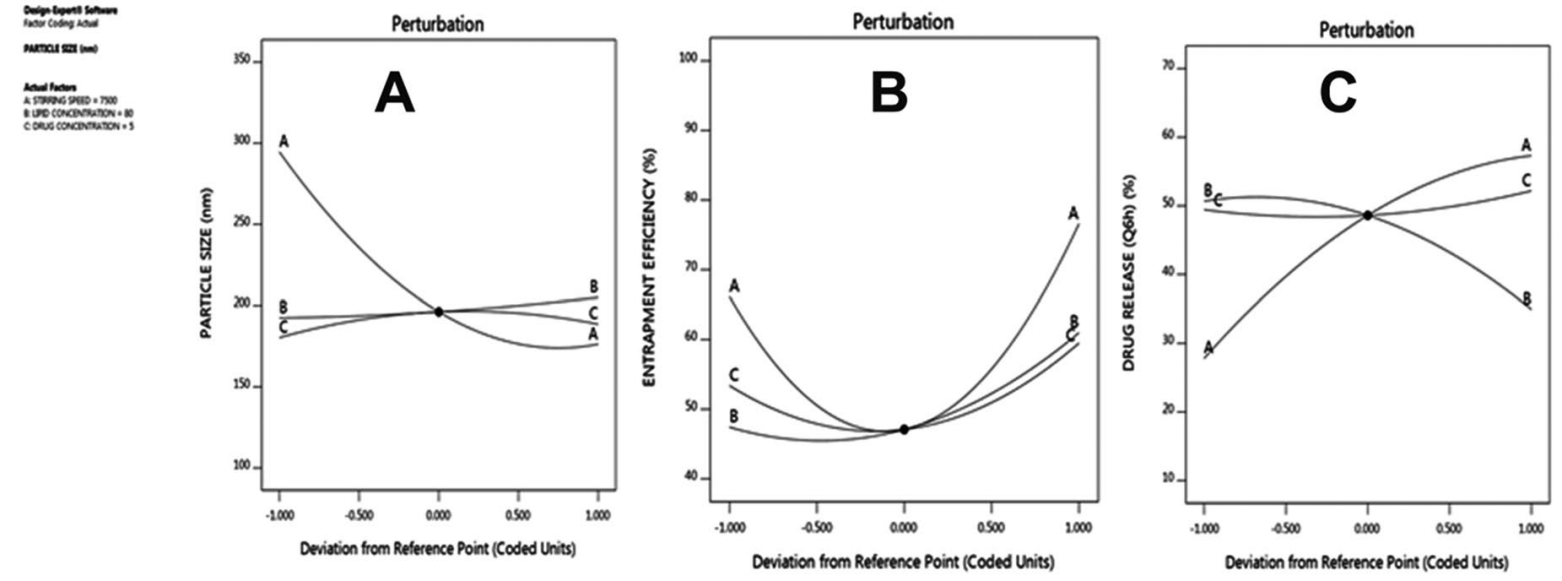
Perturbation plots showing the impact of each of the independent variables on particle size, entrapment efficiency, and drug release of NLCs.

The perturbation plots show the impact of each of the independent variables (stirring speed, lipids, and β-sitosterol) on particle size, entrapment efficiency, and drug release, see Figure 4. The result revealed that all three factors were directly involved in the NLC characters.

Table 3 shows the statistical parameters obtained from ANOVA for the reduced models, such as adjusted R2, model p-value, sufficient precision, and percent CV. With a P-value of <0.0001, particle size, entrapment efficiency, and drug release were found to be well matched to the quadratic model. According to the coefficients and P-value of the linear mixture, all of the linear mixture components (A, B, and C) were effective on the response based on the values of linear terms. All points were found to be placed in a normal distribution after evaluating the reactions of outliers. For Y1, Y2, and Y3, the ‘adjusted R2’ is in reasonable agreement with the ‘predicted R2’. The signal-to-noise ratio is measured by ‘adequate precision’. It is preferable to have a ratio of more than four. A good signal is indicated by the ratios of 10.0863 for Y1, 22.0314 for Y2, and 12.2622 for Y3. The design space can be navigated using this concept. The results demonstrated that the Box-Behnken design model could explain 90% of the response variations in particle size, entrapment effectiveness, and drug release as a function of the primary composition. We can infer that the quadratic model was a good fit for the analysis since it accurately depicted the trends, and the interaction between the parameters had a greater impact on particle size, entrapment efficiency, and drug release of nanostructured lipid carriers.

**Table 3.** ANOVA analysis for (Y1), (Y2), and (Y3) of the prepared β-sitosterol loaded NLC formulations.

Responses	Adjusted R^2^	Predicted R^2^	Model P values	Adequate precision	% CV	Model F values
Particle size (Y_1_)	0.8240	0.7417	<0.0038	10.0863	9.96	9.32
Entrapment efficiency (Y_2_)	0.9721	0.9001	<0.0001	22.0314	4.08	62.99
Drug release (Y_3_)	0.8598	0.7605	<0.0018	12.2622	11.52	11.90

### Evaluation of the optimized β-sitosterol loaded NLC formulation

An optimized and stable β-sitosterol-loaded NLC formulation has the smallest particle size, the entrapment efficiency of >80% and the drug release of >50%. According to the predetermined constraints for each independent variable, the design expert software version 11 automatically generated the optimized formula. As represented in Table 4, the optimized formulation was prepared at 9999.99 rpm stirring speed, 89.337% of lipid concentration and 5% of drug concentration.

**Table 4.** Optimum formulation derived by Box-Behnken experimental design.

Factors	Stirring speed (rpm)	Lipid concentration (%)	Drug concentration (%)	Desirability
Optimal formulation	9999.99	89.337	5	0.850

Desirability of optimum formulation was found to be 0.850 as shown in Table 4. The formulation quality was regarded as excellent and acceptable, when the desirability value was between 0.8 and 1. When this value was less than 0.63, the formulation quality was judged poor. When the desirability value was less than 0.37, the formulation performance was considered unacceptable (Masoumi et al., 2015).

The predicted values of the responses were 183.92 nm, 88.906% and 42.323% for particle size, entrapment efficiency and drug release respectively, see Table 5.

**Table 5.** Experimental and predicted values under optimal assay conditions for β-sitosterol – loaded NLC formulation.

	Particle size (nm)	Entrapment efficiency (%)	Drug release Q_6h_ (%)
Predicted	183.92 ± 3.65	88.906 ± 1.22	42.323 ± 2.6
Experimental	193.6 ± 1.52	89 ± 2.1	45.88 ± 2.8
% error	5.26	0.105	8.40

%error = (observed value – predicted value)/predicted value × 100.

The new batch of NLCs was formulated by the method prescribed before and responses were measured. The observed values of responses were compared to the predicted values and % error was calculated to validate the method. There was a very close agreement between the observed values of Y1, Y2 and Y3 and the predicted ones. By this the validity of the optimization procedure was proven.

**Figure 5.**
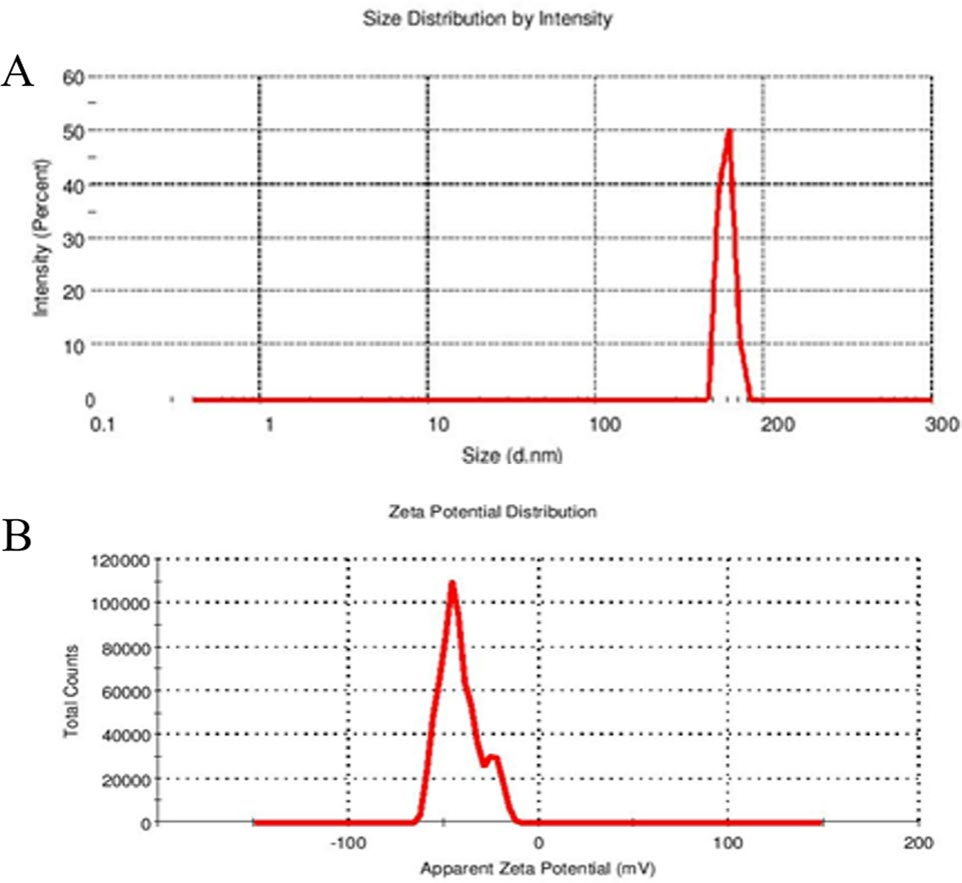
Particle size distribution and zeta potential of the optimized β-sitosterol loaded NLC formulation.

β-sitosterol loaded NLC was successfully prepared by the hot high-pressure homogenization method. As shown in Figure 5(A), the optimized formulation showed good particle size distribution, where the particle size was found to be 193.6 ± 8.23 nm. This is further supported by PDI values of 0.370 ± 0.02. PDI values within 0 and 1 indicate the uniformity of the particle size (Kumar & Dixit, 2017). Zeta potential was found to be −21.4 ± 1.76, which indicates that the surface charge was negative and the obtained results were shown in Figure 5(B). The negative charge on the surface of the optimized formulation may be attributed to the ionization of the carboxylic acid groups in coconut oil and of glyceride fatty acids in Glyceryl monostearate. It is known that Zeta potential values of more than ± 30 are considered as a good indication for the stability of the nanostructured lipid carrier (Mitri et al., 2011).

**Figure 6.**
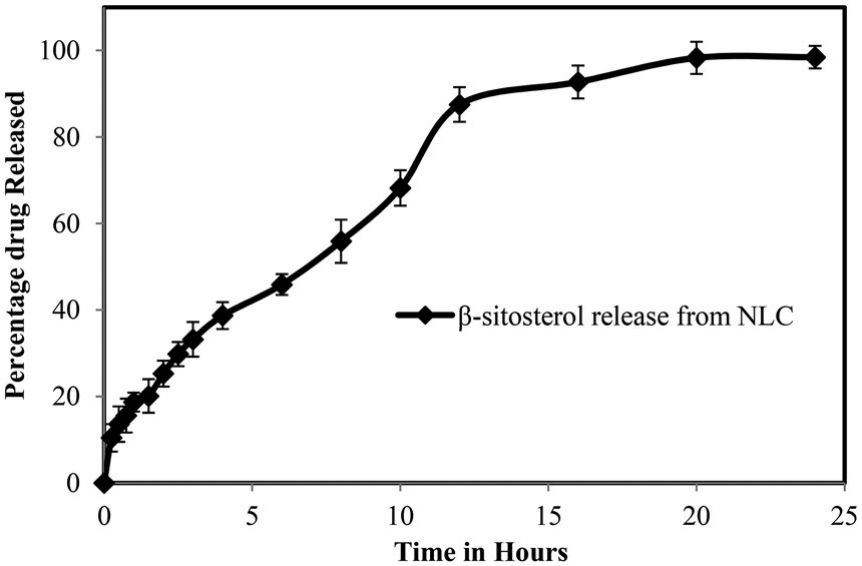
The in-vitro release profile of the optimized β-sitosterol-loaded NLCs.

The dialysis membrane method was used to study the in-vitro release of β-sitosterol from optimized NLCs in a pH 7.4 phosphate buffer, and the release profile is shown in Figure 6. In phosphate buffer, the release profiles of NLC formulation showed a typical biphasic release pattern, with an initial rapid phase followed by a gradual phase. The initial quick phase may have contributed to the desorption of the drug from the outer surface which resulted in a short diffusion path length. The solubility of the drug is improved during the formulation process when the temperature is raised to 70 °C–80 °C in the presence of a surfactant in the aqueous phase. During the cooling phase, the medication is partitioned into the lipid phase. A solid lipid core is formed when the solid lipid recrystallizes. The drug is encapsulated in the core lipid matrix and deposited on the shell and/or surface of the lipid nanoparticles in smaller amounts. As a result, the formulation has the least quantity of drug on the surface, and the outer shell of the β-sitosterol loaded NLC contributes to the initial quick release; additionally, the drug in the lipid matrix’s core contributes to the second slow release phase. Because solid lipid (GMS) has a higher melting point, liquid lipid will be randomly distributed at low temperatures. Liquid lipids (Coconut oil) would be found at the outside shell of the nanoparticles instead of being distributed throughout the solid lipid core when the liquid lipid concentration is increased. At first, this resulted in a drug-enriched shell linked with drug burst release. The crystalline structure of NLCs became more defective when liquid lipid was spread in solid lipid, allowing the drug to be released more easily, increasing the rate of drug release (Lacatusu et al., 2012). This result was further supported by Fahmy et al. (2020), who developed nanostructured lipid carrier (NLC), using Compritol® 888 and coconut oil and found that a biphasic release pattern during in-vitro drug release study (Fahmy et al., 2020).

Several kinetic models were used to analyze the release curves, with the Korsmeyer–Peppas model providing the best fit (r2 = 0.986). ‘n’ was found to have a value of 0.54. ‘n’ values between 0.43 and 1 imply an atypical, non-Fickian transport, according to the Korsmeyer-Peppas model. This suggests that the release of β-sitosterol was driven by two mechanisms: an initial ‘burst’ release caused by the non-encapsulated substance, and a sustained release regimen determined by the fraction of β-sitosterol loaded by the NLC.

### The surface morphology of the optimized β-sitosterol loaded NLC formulation (SEM)

**Figure 7.**
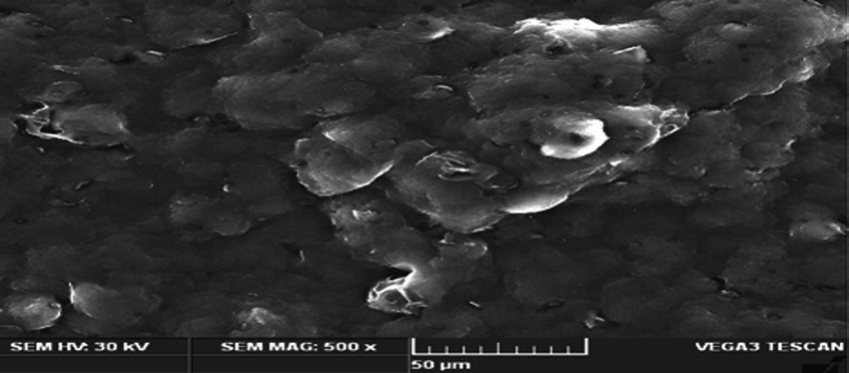
SEM images of the optimized β-sitosterol loaded NLC formulations.

SEM image of the optimized NLC was shown in Figure 7, the particles appeared spherical with smooth surface. The findings were in agreement with previous report; smaller particles increased the skin permeation of drugs by enhanced adhesion and occlusion compared to larger particles (Choi et al., 2010).

### Evaluation of NLC-MN

**Figure 8.**
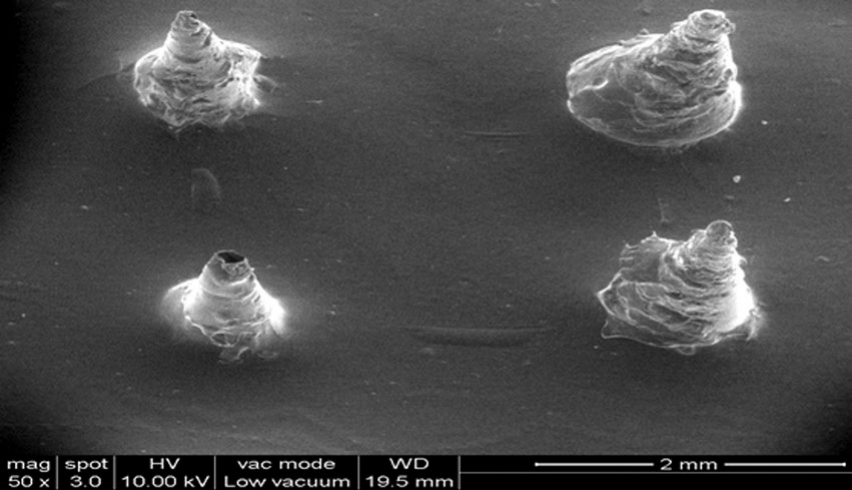
β-sitosterol loaded NLC-MN formulation.

Generally, nanocarriers can’t reach the deep layers of skin easily. This indicates that a low amount of drug delivery would not necessarily target the deeper tissues. But micro puncturing the skin using MNs will enable the delivery of drugs to deep dermal layers. Chitosan was selected as a bio-erodible hydrophilic polymer to synthesize the MNs and β-sitosterol-loaded NLCs were incorporated into the chitosan-based MNs to prepare NLC-MNs. The physical properties and image of the prepared chitosan-based MNs was shown in Table 6 and Figure 8, respectively.

**Table 6.** Physical characteristics of the Microneedles.

Physical characteristics (n = 20)	
Length (μm)	504.12 ± 22.03
Base diameter (μm)	413.01 ± 25.76
Tip diameter (μm)	31.33 ± 5.28
Aspect ratio	0.96 ± 0.11
Drug entrapment (n = 3)	92.14 ± 3.58

Values are the mean ± standard deviation.

One of the crucial characteristics that determines the MNs’ insertion capacity through the skin is the aspect ratio, which is the ratio of length to base diameter. The aspect ratio of the NLC-MNs employed in this work was substantially lower than that of conical polymeric MNs, indicating that they were short. The lower the MN’s aspect ratio, the more difficult it is to penetrate the skin (Lee et al., 2014).

**Figure 9.**
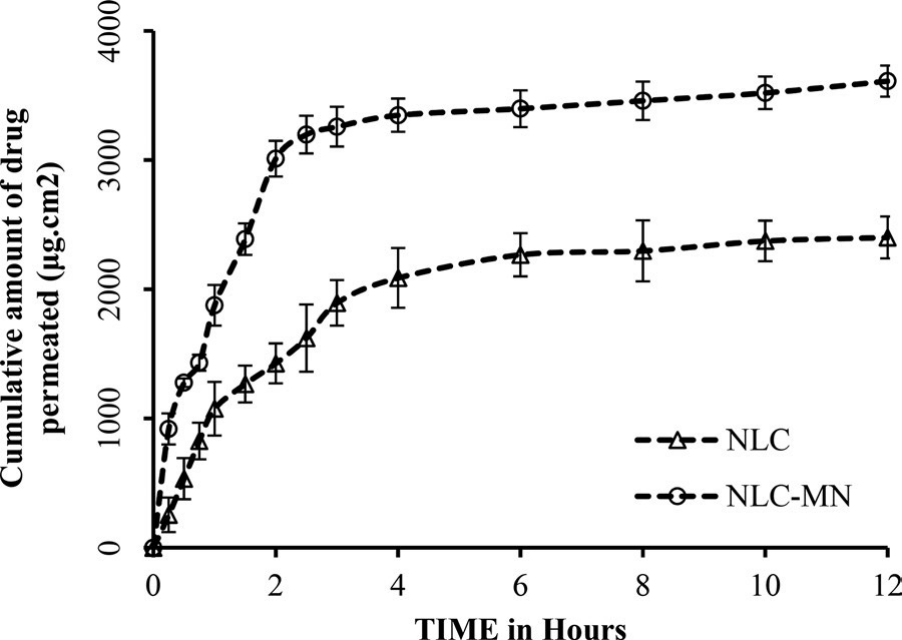
Ex-vivo skin permeation of NLC and NLC-MN formulation.

In this study, NLC- MN formulations improved the transdermal penetration of β-sitosterol effectively, when compared to that of NLC preparation (Figure 9). The improvement of transdermal drug delivery is due to the MN’s ability to overcome the barrier properties of the stratum corneum. The results showed that the cumulative amount of β-sitosterol associated with NLC- MN permeated was 3612.27 ± 120.81 μg/cm^2^, while from the NLC preparation was 2402.35 ± 162.5 μg/cm^2^. The steady-state flux (J_ss)_ of NLC-MN was significantly (*P* < 0.05) higher than that of the NLC formulation. This result revealed that β-sitosterol-loaded NLCs incorporated in MNs achieved an enhanced in-vitro percutaneous permeation rate than that observed with the plain NLC drug delivery system.

Because these microneedles formed micropassages, the mechanism was a combination of diffusion via the skin membrane and mass transfer through micron-sized channels across skin, indicating that it is not a pure membrane diffusion process (Kochhar et al., 2012). When comparing conventional skin permeation, a steady state can be generated by following Fick’s first law for membrane diffusion, which states that the donor concentration remains constant during the diffusion. However, during the permeation process, the donor concentration reduced in this investigation because microneedles let the drug diffuse faster.

The release of active medicinal substances from dissolving MN arrays is primarily governed by the dissolution of the MN matrix (Prausnitz, 2004; Donnelly et al., 2012). These findings were consistent with a prior study that described the use of nanoparticles in combination with MNs to improve medication penetration and deposition in the upper layer of the skin (Waghule et al., 2019). After application, NLC-MNs quickly dissolved in the extracellular fluid of the skin, and medication was released from the liberated NLCs in a regulated manner, finally reaching the target site. The NLCs diffused and/or partitioned into the appropriate dermal layer during this step, delivering the medication at a prolonged rate.

### In vivo efficacy of NLC-MN

After a 21-day treatment with testosterone, a patch of hair loss from the dorsal region of the rat was clearly visible in the animals of group B. The animals treated with β-sitosterol showed less hair loss after 21 days than those treated with testosterone, according to ocular observations. Table 7 shows that the hair growth pattern in NLC-MN treated groups was equivalent to that in β-sitosterol loaded NLC and gel formulations.

**Table 7.** Comparison of hair growth pattern in NLC-MN treated groups to β-sitosterol loaded NLC and gel formulations.

Treatment group	Follicular density (no./mm)	Anagen	Telogen	Anagen/telogen ratio
(A) Intact control (did not receive testosterone)	1.43 ± 0.20	65.6 ± 4.8	31.9 ± 3.2	2.06 ± 0.61
(B) Testosterone solution only	0.81 ± 0.30*	20.3 ± 3.1*	79.6 ± 4.6*	0.26 ± 0.08*
(C) Testosterone with β-sitosterol gel	1.06 ± 0.12**	31.7 ± 4.0**	81.9 ± 5.1**	0.39 ± 0.25**
(D) Testosterone + NLCs	1.95 ± 0.25**	71.8 ± 3.9**	52.1 ± 4.8**	1.24 ± 0.18**
(E) Testosterone + NLC-MNs	2.74 ± 0.32***	87.6 ± 2.7***	39.4 ± 3.6***	2.22 ± 0.34***

Values represent mean ± SD.

* *p* < 0.005, significance vs control.

** *p* < 0.05, significance vs testosterone (s.c.).

*** *p* < 0.05, significance of NLC-MN compared to NLC.

The follicular density in the NLC and NLC-MN treated groups was 2.74 ± 0.32 and 2.49 ± 0.25, respectively, but in testosterone-treated animals it was 0.810.30. Based on the cyclic phase of hair follicles, the anagen/telogen ratio was computed (anagen, telogen). The mice treated with the β-sitosterol gel had fewer hair follicles in the telogen phase than in the anagen phase. The number of hair follicles in the anagen phase was substantially higher in the group treated with the β-sitosterol laden NLC than in the telogen phase. When the NLC-MN treated group was studied, it was discovered that the number of hair follicles in the anagen phase was highest, while the number of hair follicles in the telogen phase was lowest.

Anagen/telogen ratio was significantly affected by NLC and NLC-MN, which was 2.22 ± 0.34, 1.24 ± 0.18 respectively as against 0.26 ± 0.08 for testosterone-treated animals. The reversal of androgen-induced hair loss in β-sitosterol -treated mice is indicated by the prevalence of hair follicles in anagenic growth phase. The NLC–MN delivery system, on the other hand, was proven to significantly improve hair growth in rats.

**Figure 10.**
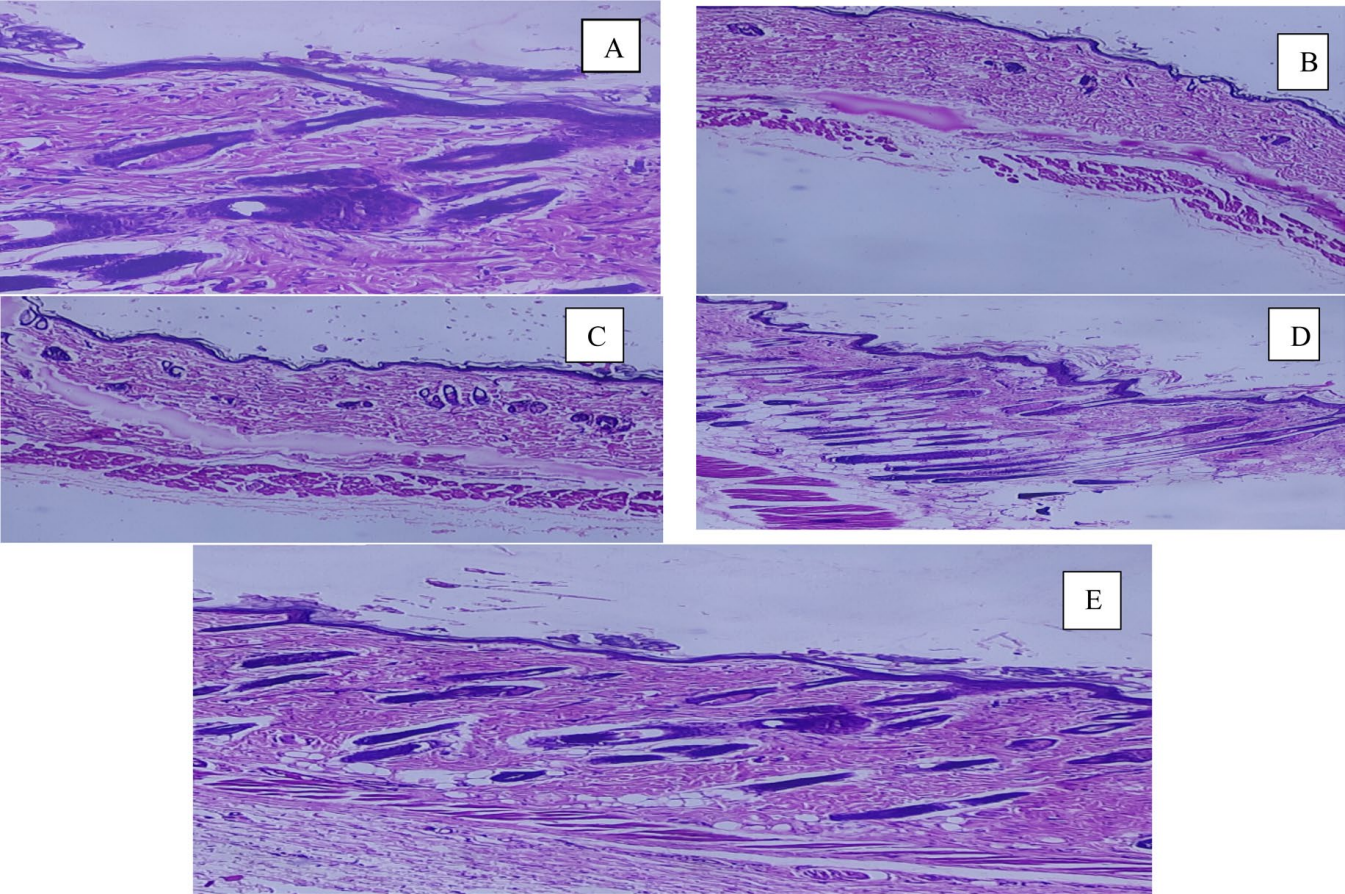
Histology of skin sections Images (A) Control; (B) Testosterone solution only; (C) Testosterone with β-sitosterol gel; (D) Testosterone + NLCs; (E) Testosterone + NLC-MNs.

The measurement of hair growth was depicted in Figure 10.

Testosterone induced alopecia, was counteracted when the formulation contains β-sitosterol and coconut oil was administered topically simultaneously to the rats by inhibiting the synthesis of DHT, which is considered to be the main cause of hair loss. In the hair follicles, usually the androgen exerts its effect either directly or after conversion by the enzyme, 5α-reductase, to dihydrotestosterone, which is a more potent androgen that binds to androgen receptor in hair follicles (Prager et al., 2002). Arruzazabala et al. (2007) found that coconut oil inhibits prostate 5α-reductase activity as it contains high content of lauric and myristic acids (Arruzazabala et al., 2007). Repeated microneedling stimulation enhanced hair growth via activation of the hair growth related genes Wnt/β-catenin and platelet derived growth factor and stem cells (Jeong et al., 2012; Kim et al., 2012). Lee et al. (2014) suggested that the minimal invasion of the skin conferred by the polymeric microneedles could provide channels for delivering the drug with high efficiency in a controlled or sustained release pattern across the skin (Lee et al., 2014). The NLC embedded chitosan microneedles may serve as a reservoir, slowly releasing the β-sitosterol at the insertion site and enhancing drug concentration. The swelling and gradual degradation of the chitosan microneedles is attributed to this extended release.

#### Skin irritation test

The skin irritation score (erythema and edema) of the β-sitosterol loaded NLC and NLC-MN formulations was less than 2 in the skin irritation test (Table 8). Formulations that produce a score of 2 or less are judged negative (no skin irritation) as mentioned by Draize (1944). As a result, NLC-MN has no skin irritation and is biocompatible. As a result, even long-term interaction with transdermal patches or adhesives will not irritate the skin.

**Table 8.** Skin Irritation Scores Following Transdermal NLC-MN Administration.

	Control		NLC		NLC-MN		Formalin	
Rat No	Erythema^#^	Edema^$^	Erythema^#^	Edema^$^	Erythema^#^	Edema^$^	Erythema^#^	Edema^$^
1	0	0	1	0	1	1	3	3
2	0	0	0	1	2	1	3	2
3	0	0	1	1	1	0	3	2
4	0	0	1	1	2	1	2	2
5	0	0	1	0	1	1	3	3
6	0	0	1	0	2	1	3	3
Average			0.83 ± 0.41*	0.50 ± 0.55*	1.50 ± 0.55*	0.83 ± 0.41*	2.83 ± 0.41	2.50 ± 0.55

# Erythema scale: 0, none; 1, slight; 2, well defined; 3, moderate; and 4, scar formation.

$ Edema scale: 0, none; 1, slight; 2, well defined; 3, moderate; and 4, severe.

* Significant compared with formalin groups (*P* < 0.05).

## Conclusion

The transdermal delivery of β-sitosterol increasing the local concentration of active molecules has received considerable attention in topical applications. MN product could be successfully developed based on the technology and data presented here and easy scaling up NLC-MN patch is a relatively straightforward process. Commercially, β-sitosterol loaded NLC-MN is considered as a useful preparation to be used topically in androgenic alopecia and other androgen related disorders.

## Data Availability

Available on request.
